# A machine learning based framework to identify and classify long terminal repeat retrotransposons

**DOI:** 10.1371/journal.pcbi.1006097

**Published:** 2018-04-23

**Authors:** Leander Schietgat, Celine Vens, Ricardo Cerri, Carlos N. Fischer, Eduardo Costa, Jan Ramon, Claudia M. A. Carareto, Hendrik Blockeel

**Affiliations:** 1 Department of Computer Science, KU Leuven, Leuven, Belgium; 2 Department of Public Health and Primary Care, KU Leuven Kulak, Kortrijk, Belgium; 3 Department of Respiratory Medicine, Ghent University, and VIB Inflammation Research Center, Ghent, Belgium; 4 Department of Computer Science, UFSCar Federal University of São Carlos, São Carlos, São Paulo, Brazil; 5 Department of Statistics, Applied Mathematics, and Computer Science, UNESP São Paulo State University, Rio Claro, São Paulo, Brazil; 6 Instituto de Ciências Matemáticas e de Computação, Universidade de São Paulo, São Carlos, São Paulo, Brazil; 7 INRIA Lille Nord Europe, 40 avenue Halley, 59650 Villeneuve d’Ascq, France; 8 Department of Biology, UNESP São Paulo State University, São José do Rio Preto, São Paulo, Brazil; Rutgers University, UNITED STATES

## Abstract

Transposable elements (TEs) are repetitive nucleotide sequences that make up a large portion of eukaryotic genomes. They can move and duplicate within a genome, increasing genome size and contributing to genetic diversity within and across species. Accurate identification and classification of TEs present in a genome is an important step towards understanding their effects on genes and their role in genome evolution. We introduce TE-Learner, a framework based on machine learning that automatically identifies TEs in a given genome and assigns a classification to them. We present an implementation of our framework towards LTR retrotransposons, a particular type of TEs characterized by having long terminal repeats (LTRs) at their boundaries. We evaluate the predictive performance of our framework on the well-annotated genomes of *Drosophila melanogaster* and *Arabidopsis thaliana* and we compare our results for three LTR retrotransposon superfamilies with the results of three widely used methods for TE identification or classification: RepeatMasker, Censor and LtrDigest. In contrast to these methods, TE-Learner is the first to incorporate machine learning techniques, outperforming these methods in terms of predictive performance, while able to learn models and make predictions efficiently. Moreover, we show that our method was able to identify TEs that none of the above method could find, and we investigated TE-Learner’s predictions which did not correspond to an official annotation. It turns out that many of these predictions are in fact strongly homologous to a known TE.

## Author summary

Over the years, with the increase of the acquisition of biological data, the extraction of knowledge from this data is getting more important. To understand how biology works is very important to increase the quality of the products and services which use biological data. This directly influences companies and governments, which need to remain in the knowledge frontier of an increasing competitive economy. Transposable Elements (TEs) are an example of very important biological data, and to understand their role in the genomes of organisms is very important for the development of products based on biological data. As an example, we can cite the production biofuels such as the sugar-cane-based ones. Many studies have revealed the presence of active TEs in this plant, which has gained economic importance in many countries. To understand how TEs influence the plant should help researchers to develop more resistant varieties of sugar-cane, increasing the production. Thus, the development of computational methods able to help biologists in the correct identification and classification of TEs is very important from both theoretical and practical perspectives.

## Introduction

Transposable elements (TEs) are DNA sequences that can move and duplicate within a genome, autonomously or with the assistance of other elements. The field of TE annotation includes various steps such as the identification and classification of TE families. In this article, we focus on these activities since accurate identification and classification of TEs enable researches into their biology and can shed light on the evolutionary processes that shape genomes [1].

**Fig 1:**
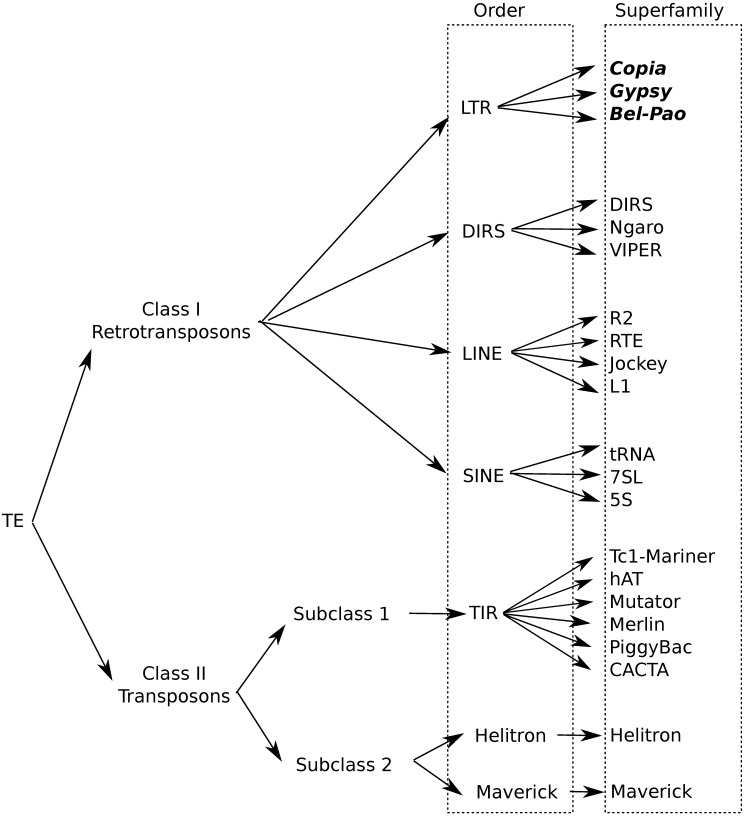
Copia, Gypsy and Bel-Pao superfamilies positioned in Wicker’s taxonomy [2].

TEs in eukaryotes can be classified according to whether reverse transcription is needed for their transposition (Class I or retrotransposons) or not (Class II or DNA transposons). A consensus for a universal TE classification has not been reached yet [3], but this lack of consensus does not affect the focus of our study. Here, we will follow the hierarchical system proposed by Wicker et al. [2], which includes the levels of class, subclass, order, superfamily, family and subfamily. Fig 1 presents an illustration of Wicker’s hierarchy considered in our study. Class I is composed of five orders: LTR retrotransposons, DIRS-like elements, Penelope-like elements (PLEs), long interspersed nuclear elements (LINEs) and short interspersed nuclear elements (SINEs). Similar in structure to retroviruses, LTR retrotransposons have long terminal repeats (LTRs), two normally homologous non-coding DNA sequences that flank the internal coding region and that range in size from a few hundred base pairs to more than 5 kb. Superfamilies within an order are distinguished by uniform and widespread large-scale features, such as the structure of protein or non-coding domains and the presence and size of the target site duplication (TSD). Families are defined by DNA sequence conservation and subfamilies on the basis of phylogenetic data. Class II is divided into two subclasses, which are distinguished by the number of DNA strands that are cut during transposition. Subclass 1 consists of TEs of the order TIR, which are characterized by terminal inverted repeats (TIRs). Subclass 2 groups the Helitron and Maverick orders.

Methods identifying TEs in a genome are homology-based, employ structural information or do not use prior information at all about the TEs to be identified [4–6]. The latter methods, known as de novo repeat discovery methods, search for example for repeats in the genome. A widely used method for TE identification is RepeatMasker [7]. This tool screens a query sequence searching for repeats, taking into account their similarity with sequences from a reference library, using an optimal pairwise alignment algorithm. Censor [8] works similarly as RepeatMasker but uses BLAST for the comparison. Afterwards, both RepeatMasker and Censor remove overlaps and defragment detected repeats. Loureiro et al. [9] show that machine learning can be used to improve the identification of TEs. They assessed a set of (non-machine learning based) identification methods and learn a classifier that combines their predictions to determine whether a sequence is a TE or not. Another classifier predicts the best method to determine the exact boundaries of a TE. In their analysis, both RepeatMasker and Censor were the most accurate tools. While Loureiro et al. demonstrate the benefit of using machine learning models to *improve* predictions, they do not use machine learning to *obtain* the predictions, which we address in this article.

A few methods have been proposed to classify TEs. LtrDigest [10] evaluates a list of LTR retrotransposons generated by another tool called LTRharvest [11], annotating these sequences w.r.t. the protein domains and other structural characteristics that were found in them. LtrDigest can then be used for de novo (unsupervised) classification, i.e., finding groups within the LTR retrotransposons without any predefined classification scheme. To evaluate whether the resulting groups represent known LTR retrotransposon superfamilies, Steinbiss et al. [10] have matched representative sequences of the groups to a reference set of known transposon sequences using a fixed set of rules. LtrSift [12] takes the LtrDigest output and clusters the candidate sequences. It then tries to find patterns of shared cluster membership that might indicate multiple TE families, e.g. different Copia-like, Gypsy-like or Bel-Pao families. It is a generic tool that uses sequence clusters to find family-specific patterns, based on the LtrDigest detected features. These patterns are then used as evidence for family discrimination. TEClass [13] classifies TE sequences into Class I and Class II TEs. The Class I elements can further be classified into LTRs and non-LTRs, and the non-LTRs are classified into the SINE or LINE orders. This classification is obtained by a hierarchy of binary classifiers based on machine learning support vector machines, using oligomer frequencies as features. RepClass [14] consists of three independent classification modules: a module based on homology information, a module that searches for structural characteristics such as LTRs or TIRs, and a module that searches for target site duplication. The three modules provide classifications at different levels of granularity, typically at the subclass or order level, sometimes at the superfamily level. Finally, an integration module aims to compare, rank, and combine the results of the three modules providing a single tentative classification. Pastec [15] also uses multiple features of TEs to classify TE sequences: structural features (TE length, presence of LTRs or TIRs, presence of simple sequence repeats, etc.), sequence similarities to known TEs, and conserved functional domains found in HMM profile databases. It provides classifications on the order level, including all orders from the classification hierarchy defined by Wicker et al. [2], whereas TEClass and RepClass only consider a subset of the orders. Importantly, none of the above classification systems is able to provide classifications for LTR retrotransposons at the superfamily level. One exception is a recently introduced method called LtrClassifier [16], which performs both annotation (i.e., identifying structural elements) and classification (but not identification) for plant genomes, and returns predictions for the Copia and Gypsy superfamilies.

In this article we introduce TE-Learner, a framework for the identification of TEs of a particular order, and for the classification of these TEs on the superfamily level. TE-Learner consists of three steps. First, based on the characteristics of the order under consideration, it extracts from the genome a set of candidate sequences, which may include false positives. Second, it automatically annotates these candidates with features. Finally, the features are given as input to a machine learning model, which predicts whether a given candidate sequence is indeed a TE of the considered order, and if so, predicts its superfamily.

In particular, we present TE-Learner^*LTR*^, an implementation of this framework for LTR retrotransposons, which include the superfamilies Copia, Gypsy and Bel-Pao [2]. As features we consider the occurrence of conserved protein domains, which help TEs perform the transposition process. The machine learning method we apply is random forests. This last step is essential, since the model of the three superfamilies contains the same protein domains [2]; for Gypsy and Bel-Pao some domains even occur in the same order.

As LTR retrotransposons have a high abundance in the genomes of *Drosophila melanogaster* [17] and *Arabidopsis thaliana* [18, 19], and as these genomes are well annotated, they present the ideal candidates for evaluating how well our proposed method can identify and classify the LTR retrotransposons without using any prior information about these genomes. We present an extensive quantitative analysis on *D. melanogaster* and *A. thaliana* comparing the obtained results to three widely used methods (each dealing with one of the two tasks considered) and we show that TE-Learner^*LTR*^ outperforms the state-of-the-art methods w.r.t. predictive performance and runtime.

The novelty of our proposed method w.r.t. the available methods lies mainly in three aspects. First, in contrast to the other methods, which focus on one task, here we consider the tasks of identifying and classifying TEs together. Second, we propose a general framework for these tasks. Even though the implementation we provide in this article focuses on LTR retrotransposons, our framework can be extended to other TE orders. Third, in contrast to classification methods such as LtrDigest, LtrSift, RepClass, Pastec and LtrClassifier, our method is not based on a predefined set of rules. Instead, we exploit the strength of machine learning to automatically derive rules from the available data, with no need of prior knowledge. Our framework is the first step towards completely automatic identification and classification of TEs in superfamilies.

## Methods

### Framework

We address the following problem: given an unannotated genome, find subsequences in it corresponding to a particular order from the classification scheme [2], and predict their superfamily. We propose the following three-step framework, called TE-Learner:

1. The genome is split into subsequences, that become the candidate TE sequences.
2. Every candidate TE sequence is annotated with features related to the TE order considered.
3. Every candidate is represented by its features and fed into a machine learning model. The model predicts for every candidate the probability that the sequence belongs to a specific superfamily of the order considered.

**Fig 2:**
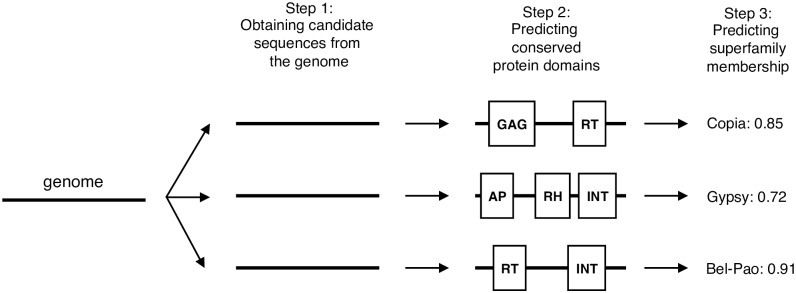
A schematic representation of our framework, applied to LTR retrotransposons.

We now discuss TE-Learner^*LTR*^, one particular implementation, for every step in detail, focusing on the LTR retrotransposon order. In Step 1 we use a simple splitting strategy to obtain subsequences of the genome. The features used in Step 2 are conserved protein domains known to occur in LTR retrotransposons, and the machine learning model used in Step 3 is a random forest. Fig 2 shows a schematic representation of our framework based on this implementation.

Note the modularity of the framework: every step can be implemented independently of the other steps. For instance, an alternative implementation could use an LTR pair detection tool in Step 1, annotate the candidates with oligomer frequencies in Step 2, and apply an artificial neural network in Step 3. Any machine learning classifier can be used, as long as it outputs a probability.

#### Step 1: Obtaining candidate LTR retrotransposons from the genome

First, the genome needs to be cut into subsequences, which together with their conserved protein domain information are used as input to the machine learning model. In our approach, we use a sliding window to generate all subsequences of a particular length (10,000 nucleotides in our tests) with an overlapping between them that avoids important regions potentially being cut (1,000 nucleotides in our tests). For this first step, one interested, for example, in potential full-length LTR retrotransposons could alternatively use existing tools [11, 20] to build the sets of candidates based on their LTR pairs.

The output of this first step is a list of potential LTR retrotransposon candidates, which will be searched for conserved protein domains (Step 2) and assigned probabilities of belonging to a particular superfamily (Step 3). Even though this first step generates a lot of false positives, the following steps will correct for these.

#### Step 2: Predicting conserved protein domains

The second step consists in screening every candidate TE, obtained previously, for the presence of conserved protein domains that can be found in LTR retrotransposons. To that aim, we use the RPS-Blast program [21]. RPS-Blast (Reverse Position-Specific Blast) uses a query sequence to search a database of pre-calculated position specific score matrices (PSSMs), built from multiple sequence alignments. In order to accelerate the search process, we constructed a database that only contains the PSSMs related to conserved protein domains found in LTR retrotransposons. More precisely, our database consists of 26 PSSMs, corresponding to the list of conserved subdomains shown in Table 1.

**Table 1:** List of 26 protein domains considered in the tests.

Protein domain	Subdomain (CDD/Pfam ID)
RNase	RNase_HI_RT_Ty1 (cd09272)
	RNase_HI_RT_Ty3 (cd09274)
	RNase_HI_like (cd09279)
	RNase_HI_RT_DIRS1 (cd09275)
Integrase	rve (pfam00665)
GAG	Retrotrans_gag (pfam03732)
	Retrotran_gag_2 (pfam14223)
	Retrotran_gag_3 (pfam14244)
	gag-asp_proteas (pfam13975)
	DUF1759 (pfam03564)
AP	retropepsin_like (cd00303)
	retropepsin_like_LTR_1 (cd05481)
	retropepsin_like_LTR_2 (cd05484)
	RP_Saci_like (cd06094)
	RVP_2 (pfam08284)
	Peptidase_A17 (pfam05380)
	DUF1758 (pfam05585)
RT	RT_LTR (cd01647)
	RT_pepA17 (cd01644)
	RVT_1 (pfam00078)
	RVT_2 (pfam07727)
	RVT_3 (pfam13456)
	RT_DIRS1 (cd03714)
Pre-integrase	gag_pre-integrase (pfam13976)
YR	INT_Cre_C (cd00799)
	DNA_BRE_C (cd00397)

The resulting domain hits are used to provide an estimation of the boundaries of the TE candidate. In particular, we considered that each candidate starts at the beginning of the first predicted domain and ends at the final position of the last predicted domain. In case a domain is found in the overlap region between two sequences, we merged these sequences before determining their boundaries. In case a candidate contains predicted domains in both DNA strands (direct and reverse), we split it in two or more new candidates. Note that our boundaries do not include the LTR regions.

To summarize, for each candidate TE sequence returned by Step 1, this second step produces a list of conserved protein domains, which is used to delineate the candidate. Finally, the candidates with at least one domain hit are passed on to Step 3.

#### Step 3: Predicting superfamily membership

**Fig 3:**
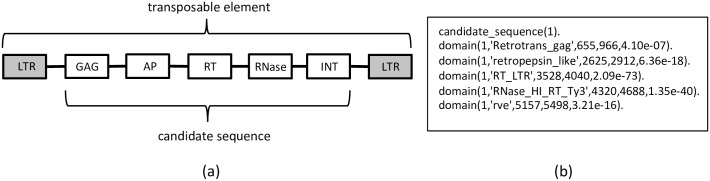
Logical representation. (a) Illustration of the typical structure of a TE from the Gypsy superfamily, delimited by LTRs and with protein domains identified. (b) An example of a candidate sequence, annotated with protein domain predictions. Each domain prediction lists the candidate ID, the domain, the predicted start and end positions in the sequence, and the e-value for the PSSM match. Note that domains may have subtypes. For example, RT_LTR is a subtype of domain RT.

In the final step of our method, we take the candidate TE sequences, represented by their predicted protein domains, and apply a random forest model on them. We use a first-order logic based format to represent candidate sequences. This format is more expressive than traditional tabular representations and can be handled by so-called relational learning systems (see [22] for more background on relational learning). Fig 3 gives an example of this type of representation: for each candidate sequence, information is included on which domains occur in it, between which positions, and how certain this information is (expressed as an e-value). With this representation, we can thus express TE sequences with a different number of domains in an elegant way.

In our approach, the learning process involves learning, for each LTR retrotransposon superfamily, a separate model that maps a sequence, represented as in Fig 3, to the probability that it belongs to that superfamily. The model is constructed using the FORF (first-order random forests) approach [23], which is implemented in the relational data mining system ACE (http://dtai.cs.kuleuven.be/ACE/). A random forest [24] is an ensemble of decision trees that classifies examples (candidate sequences in this case) by combining the predictions of each individual tree. The decision trees in a forest are constructed by resampling the training set and the feature set in the different tree induction processes. In this paper we use relational decision trees [25] to construct the random forest. The difference with the standard approach [26, 27] is that it allows to use the data representation defined above (instead of a traditional tabular format), to express background knowledge, and to use user-defined tests in the nodes of the trees. Apart from that, a standard top-down decision tree induction algorithm is applied. The tests that we allow include the following: (1) the occurrence of a particular protein domain, (2) the occurrence of a particular protein domain with a certain minimum or maximum length limit (the same domain can be predicted with different lengths), and (3) the spatial relationship between two domains. The protein domains that are considered in the tree nodes are the subdomains listed in Table 1, including the more general domains of the left column. Moreover, each test includes a minimum e-value for the domain hits, which is chosen from the following list: [1*e* − 50, 1*e* − 40, 1*e* − 30, 1*e* − 20, 1*e* − 10, 1*e* − 05, 1*e* − 02, 1*e* − 01]. The list of length limits employed is [20, 50, 100, 200, 500, 700]. Note that limiting the tests to individual domains or pairs of domains (rather than focusing on the complete TE domain structure) enables the classification of incomplete TEs as well. A final type of tests that are allowed in the tree nodes is (4) whether the number of occurrences of general domains (left column of Table 1) exceeds a number between 1 and 5. As such, the total number of tests considered at each tree node is 10,947.

**Fig 4:**
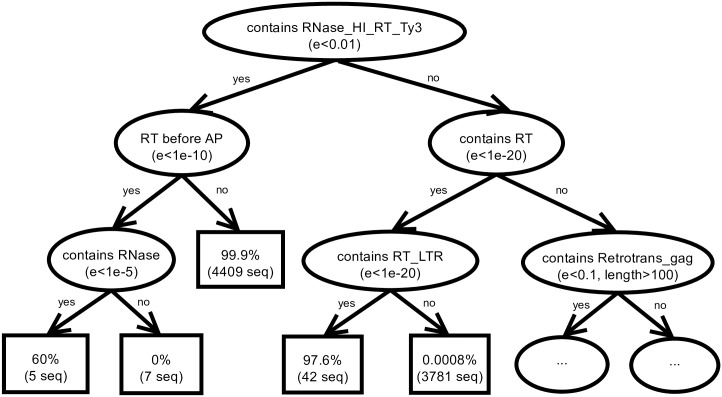
Decision tree. (Partial) decision tree predicting the probability that a given sequence belongs to the Gypsy superfamily. The abbreviation *e* in the nodes stands for e-value, *length* for the number of base pairs, *seq* for the number of training sequences reaching the leaf.

As an illustration, Fig 4 shows the (partial) relational decision tree obtained when constructing a single tree instead of a random forest for the Gypsy superfamily. Each internal (oval) node of the tree contains one of the tests defined above, and is used to route down the sequence according to the outcome of the test. The root node, in Fig 4, tests whether the sequence has a domain RNase_HI_RT_Ty3 with an e-value of less than 0.01. If it does, the sequence moves down to the “yes” branch of the tree; otherwise it moves to the “no” branch. This procedure is repeated until the sequence arrives in a leaf (rectangle) node, which provides the probability for belonging to Gypsy. The tree shows that not all Gypsy instances are discovered by merely checking the occurrence of the RNase_HI_RT_Ty3 key domain (consider the leaf node with 42 training sequences: 97.6% of them are Gypsy elements, although they followed the “no” branch at the root).

As we can see in Table 1, domains have subdomains; thus, we provide the hierarchical “is a subdomain” relationship as background knowledge to the system. This allows the tests to check for the occurrence of both domains or subdomains, even though the data representation only contains subdomains. For example, in the tree in Fig 4 there is a test that checks for the occurrence of the domain RT and tests that check for the occurrence of its subtypes (RT_LTR, for example).

TE-Learner is available for download at http://dtai.cs.kuleuven.be/software/te-learner.

### Methodology and parameter settings

We evaluate the predictive performance of our framework on the genomes of *D. melanogaster* and *A. thaliana*. We use version 6-15 of the annotated genome from Flybase (http://flybase.org/), as the official annotation for *D. melanogaster*, which was made publicly available in April 2017. We use the Flybase annotations “Transposable Elements” and “Repeat Regions” to constitute the golden standard in our experiments. For *A. thaliana*, we used the Araport11 annotation, released in June 2016, for genome TAIR10, from The Arabidopsis Information Resource (TAIR) (http://www.arabidopsis.org).

We will compare our results for the Copia, Gypsy and Bel-Pao superfamilies (Bel-Pao only for *D. melanogaster* because there is no annotation for it in *A. thaliana*) with those of three methods for TE identification or classification that can make predictions at the superfamily level: RepeatMasker, Censor and LtrDigest. For each superfamily, we also compare the results to those of a baseline model. We now discuss the specific parameter settings for each of the tools used in our framework, as well as for the methods we compare to.

**Baseline:** The baseline model starts from the TE candidates obtained in Step 2 of our framework and makes predictions solely based on the presence of one key protein domain (as predicted by the RPS-Blast program): RNase_HI_RT_Ty1, RNase_HI_RT_Ty3, and RT_pep_A17 for Copia, Gypsy, and Bel-Pao, respectively. As such, it evaluates the impact of the machine learning aspect (step 3) in our framework.

**RPS-Blast:** We constructed the database used by RPS-Blast by taking for each domain of interest the set of sequences from the Conserved Domain Database (CDD) (http://www.ncbi.nlm.nih.gov/Structure/cdd/cdd.shtml) [28], used to generate the original multiple sequence alignment, except that we excluded the sequences of the organisms used in our tests (*D. melanogaster* and *A. thaliana*, respectively). The reason to exclude the *D. melanogaster* or *A. thaliana* sequences is that we want to provide an evaluation as blindly as possible, without using any known information from the target organism. The PSI-Blast (Position-Specific Iterative Blast) program was used to obtain the new PSSMs and Makeprofiledb application for creating the database for RPS-Blast.

**FORF:** The relational trees were built with default parameters, except for the minimum number of examples in a leaf, which was set to 5. No pruning was used. The forests consist of 100 trees, with a feature sample size at each node equal to the square root of the number of possible features.

For the training of FORF we used sequences from Repbase (http://www.girinst.org/server/RepBase/), volume 17—issue 3, one set for each superfamily of interest here. In order to provide a fair evaluation, as before, we excluded from these sets the sequences of the target organism. Note that each analysis was performed twice: each time leaving out one target organism. The resulting sets were also used as the databases for RepeatMasker and Censor applications—as described further. We ran the RPS-Blast program, with the PSSM database created in Step 2 of our framework, to search these sequences for regions related to the conserved domains of interest (Table 1), retrieving the same types of information obtained from the screening of the candidates (Step 2). We observed that the longest predicted domain region in these sequences has a length smaller than 800 nucleotides, which indicates that the overlap size of 1,000 nucleotides we used in Step 1 of our framework, is sufficient. We removed training sequences without domain hits and those that contained domain hits in both strands of their genomic sequence. The resulting number of sequences is 3188 for Copia, 4718 for Gypsy, and 891 for Bel-Pao when leaving out *D. melanogaster* sequences. Leaving out *A. thaliana*, the numbers become 3077 for Copia and 4728 for Gypsy. These sequences constitute the positive training set. For each superfamily, we also constructed a negative set, by sampling without replacement from the other superfamilies. For Copia and Bel-Pao the negative set has an equal size as the positive set; however for Gypsy, given the size of its positive set, the negative set contains less sequences (all Copia and Bel-Pao sequences), which still yields a balanced classification task.

**RepeatMasker and Censor:** These systems were run using their standard parameter settings. For a fair evaluation, we used as reference library the same training sets as for FORF as described above. As each of the training sets belongs to a particular superfamily, we can label hits with the corresponding superfamily. Both applications were run on the complete genomes.

**LtrDigest:** This method was also run with its standard parameter settings on the complete genomes. We only retained predictions with an assigned DNA strand and used the authors’ guidelines to assign a particular superfamily to each prediction as follows. Every predicted sequence is annotated with protein domain hits. If the sequence has a “Peptidase_A17” hit it is classified as BelPao; otherwise, if the sequence has a “Gypsy” hit, it is classified as Gypsy; otherwise, following [10], if the sequence has an “INT” followed by an “RT” (there may be other hits in between), it is classified as Copia and if an “RT” is followed by an “INT”, it is classified as Gypsy. The remaining sequences are not classified.

### Evaluation methodology

We report the predictive performance of the different methods with precision-recall (PR) curves [29]. The motivation for preferring PR curves over the more popular ROC curves is as follows. Only a small fraction of the genome contains TE sequences of a specific superfamily, thus we are more interested in recognizing the positive cases, i.e. the candidate sequences that actually belong to the superfamily, than in correctly predicting the negatives. Precision is the percentage of predictions that are correct and recall is the percentage of annotations that were predicted. A PR curve plots the precision of a model as a function of its recall. Assume the model predicts the probability that a new example is positive, and that we threshold this probability with a threshold *t* to obtain the predicted class (positive or negative). A given threshold corresponds to a single point in PR space, and by varying the threshold we obtain a PR curve: while decreasing *t* from 1.0 to 0.0, an increasing number of examples is predicted positive, causing the recall to increase whereas precision may increase or decrease (with normally a tendency to decrease). A domain expert can choose the threshold corresponding to the point on the curve that looks most interesting.

To consider a prediction as a true positive, we do not require it to match the exact same boundaries of the corresponding annotation of the genome, as this would be an overly strict criterion. Instead, we allow some tolerance by defining a true positive as a prediction which has a minimum overlap of 100 nucleotides with an annotation, or a prediction which overlaps a complete annotation and vice versa. Our motivation for this evaluation is that a domain expert can inspect each prediction and determine the exact boundaries of the complete TE.

### Combined predictions

The random forests in TE-Learner^*LTR*^ only make predictions w.r.t. the superfamily for which they were built. For example, one forest outputs the probability whether a sequence belongs to Copia or not. However, one might be interested in having a model that can make predictions w.r.t. many superfamilies at the same time. An advantage of such a model is that the user does not need to combine the results of individual models, avoiding conflicting predictions.

As our models output probabilities, one straightforward idea to obtain this more general model consists of selecting the superfamily with the highest probability. To avoid that a superfamily with a very low probability is predicted, we include the category *None* (i.e., the sequence does not belong to any of the considered superfamilies), which is predicted when none of the probabilities exceeds a certain threshold.

In this setting, we construct a single average PR curve for all superfamilies together as follows. When a sequence is predicted to have a certain superfamily, we consider it correct if the sequence indeed belongs to that superfamily. The definition of precision and recall is then as before. Thus, for precision, the denominator contains all candidate predictions, minus those predicted as *None*; for recall, the denominator contains all annotations (for all considered superfamilies).

We compare our results to those of LtrDigest, which is also able to make predictions w.r.t. different superfamilies at the same time.

## Results

In Step 1 and 2 of our framework, we generated 3372 possible candidates for *D. melanogaster* and 2141 candidates for *A. thaliana*. In the third step, we constructed a random forest for each organism (i.e., leaving out sequences of this genome from the training data) and each superfamily. Table 2 shows the average number of nodes per tree, the training set accuracy for the forest, and the induction time. The latter shows the number of seconds needed to construct the entire forest on a MacBook Pro, 2.8 GHz Intel Core i5. With respect to the tests in the nodes of the trees, for all generated forests, we observed around 65% of the nodes testing for the occurrence of a domain with a certain length condition, around 20% without length condition, and around 15% checking the occurrence of one domain before an other. Interestingly, the number of occurrences of a particular domain did not show up in the forests.

**Table 2:** Average number of nodes per tree, training set accuracy, and induction times for the random forests.

Target organism	*D. melanogaster*			*A. thaliana*	
Superfamily	Copia	Gypsy	Bel-Pao	Copia	Gypsy
nodes	14.63	22.97	8.99	13.45	20.91
training acc.	99.65%	99.59%	99.61%	99.71%	99.59%
induction time	248.98s	354.23s	72.55s	186.33s	320.16s

We first evaluate the ability to predict each superfamily independently and then we evaluate the combined predictions, i.e., how well our framework performs in classifying a TE as Copia, Gypsy or Bel-Pao.

### Predicting each superfamily independently

Before discussing each superfamily in detail, we first show for both genomes the number of predictions that were made and the average prediction length of each method and for each superfamily (Table 3). Note that TE-Learner^*LTR*^ presents the same numbers of candidates and average length of candidates for the three superfamilies. This happens because in our implementation Steps 1 and 2 output one common candidate set for the three superfamilies. From the table it is clear that RepeatMaskerand Censor make a lot of predictions, which are on average much smaller than the predictions of TE-Learner^*LTR*^. LtrDigest on the other hand, makes much less predictions, which are considerably longer.

**Table 3:** Number of predictions and average prediction length for each organism, method and superfamily.

	*D. melanogaster*		*A. thaliana*	
Superfamily	# pred.	avg. length (bp)	# pred.	avg. length (bp)
TE-Learner^*LTR*^				
Copia	3372	2387.25	2141	1779.67
Gypsy	3372	2387.25	2141	1779.67
Bel-Pao	3372	2387.25	–	–
RepeatMasker				
Copia	3662	222.25	5317	406.25
Gypsy	27188	415.63	11987	479.63
Bel-Pao	5176	498.78	–	–
Censor				
Copia	30702	77.60	48055	99.80
Gypsy	55645	182.35	61414	125.77
Bel-Pao	8068	300.44	–	–
LtrDigest				
Copia	43	4984.53	117	5196.29
Gypsy	441	6860.89	25	6433.72
Bel-Pao	152	8120.15	–	–

#### Copia

**Fig 5:**
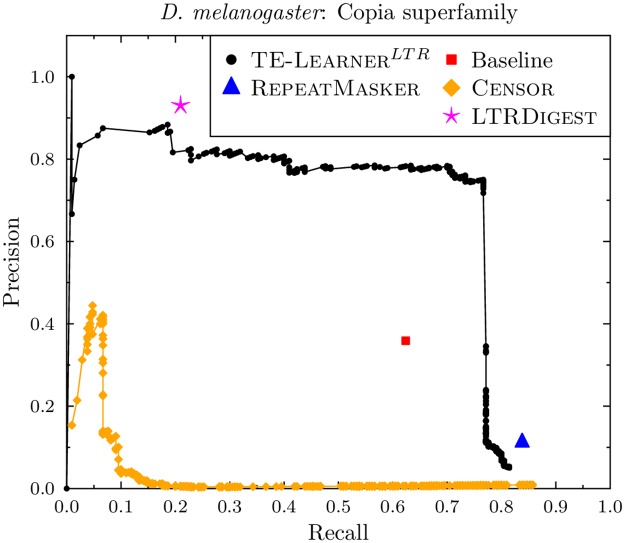
Precision-recall curves for the Copia superfamily (*D. melanogaster*).

Let us first look at the curves for *D. melanogaster* (Fig 5). First, we observe that the curve for TE-Learner^*LTR*^ has a maximal recall of 0.81. This is due to the candidate set returned by Step 2 of our method: the candidate set contains 171 of the 210 Copia TE sequences described in the annotations of *D. melanogaster*. Second, as expected, we observe that the precision of TE-Learner^*LTR*^’s curve is high for high thresholds and goes down as the thresholds are lowered. Third, RepeatMasker, LtrDigest and the baseline model only output (positive) predictions with 100% confidence rather than giving probabilities. Therefore, they correspond to a single point in PR space. While the point of the baseline model is clearly below the curve of TE-Learner^*LTR*^, the points of RepeatMasker and LtrDigest have a slightly higher value in either recall or precision, respectively, compared to TE-Learner^*LTR*^. Fourth, the curve of Censor is below the one of TE-Learner^*LTR*^, obtaining a maximal precision of 0.43, which quickly drops down close to zero. It does, however, obtain a higher recall than the other methods, with a maximum value of 0.86 (at a precision of 0.01).

**Fig 6:**
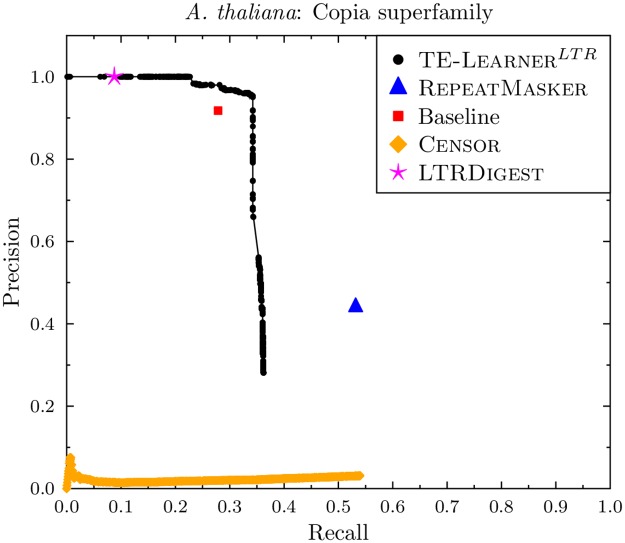
Precision-recall curves for the Copia superfamily (*A. thaliana*).

The curve for *A. thaliana* can be found in Fig 6. The recall values for *A. thaliana* are lower than the ones for *D. melanogaster*, with TE-Learner^*LTR*^ obtaining a maximal recall of 0.36, while Censor obtains a maximal recall of 0.54, and RepeatMasker reaches a recall of 0.53. Both LtrDigest and TE-Learner^*LTR*^ are able to reach a precision of 1, at a recall of 0.09 and 0.23, respectively. As before, Censor obtains a very low precision, and the baseline point is below TE-Learner’s curve.

#### Gypsy

**Fig 7:**
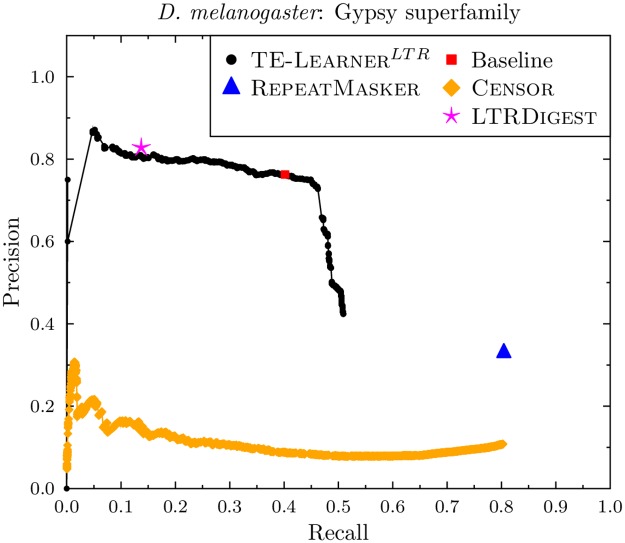
Precision-recall curves for the Gypsy superfamily (*D. melanogaster*).

Fig 7 shows the results for *D. melanogaster*. As for Copia, we find that the candidate sequences returned by TE-Learner^*LTR*^ only contain a subset of the known Gypsy sequences in *D. melanogaster* (1707 of the 3353 known Gypsy sequences). This results in a curve with a maximal recall of 0.51. The points of Censor and RepeatMasker have a lower precision than the points on TE-Learner^*LTR*^’s curve. However, both are able to obtain a higher recall (0.80). The point of LtrDigest obtains a slightly higher precision (0.83) than TE-Learner^*LTR*^ (0.81) at a recall of 0.14. However, TE-Learner^*LTR*^ is able to increase the recall considerably without giving up on precision. Interestingly, the baseline model, which scans the candidate sequences for the presence of the RNase_HI_RT_Ty3 protein domain, is situated exactly on the curve of TE-Learner^*LTR*^. This shows that this protein domain is highly predictive for Gypsy.

**Fig 8:**
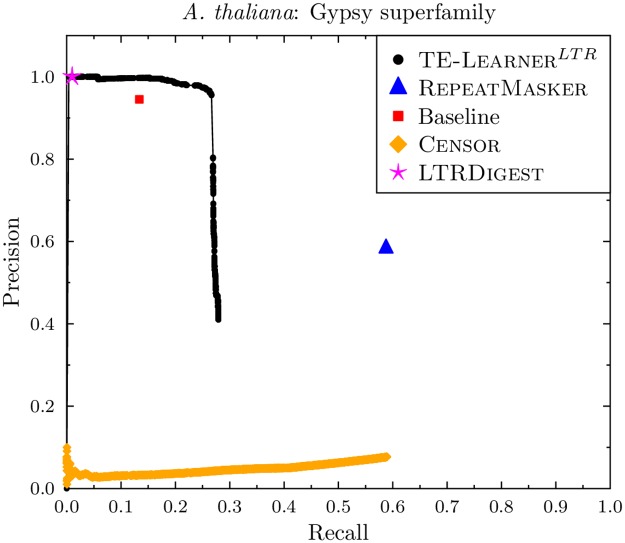
Precision-recall curves for the Gypsy superfamily (*A. thaliana*).

The curve for *A. thaliana* can be found in Fig 8. Apart from the lower recall for all methods, and the baseline point being below the TE-Learner curve, the same conclusions as for *D. melanogaster* hold.

#### Bel-Pao

**Fig 9:**
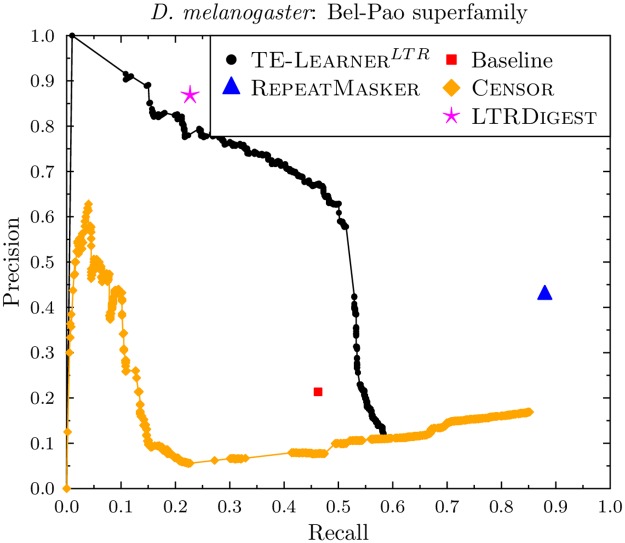
Precision-recall curves for the Bel-Pao superfamily (*D. melanogaster*).

Fig 9 shows the results for Bel-Pao. Here, our candidate sequences contain 349 of the 599 known Bel-Pao sequences in *D. melanogaster*, so the curve of TE-Learner^*LTR*^ has a maximal recall of 0.58. Its curve is above the point of the baseline model. As for Gypsy, the point of RepeatMasker and the curve of Censor are able to reach a higher recall (0.88 and 0.85, resp.) than TE-Learner^*LTR*^, while the latter obtains a much higher precision at lower recall values. LtrDigest’s point is slightly above the curve of TE-Learner^*LTR*^, obtaining a precision of 0.87 at recall 0.23, while TE-Learner^*LTR*^ obtains a precision of 0.79 at the same recall. TE-Learner^*LTR*^ does however obtain a precision of 0.89 at a recall of 0.15.

### Combined predictions

**Fig 10:**
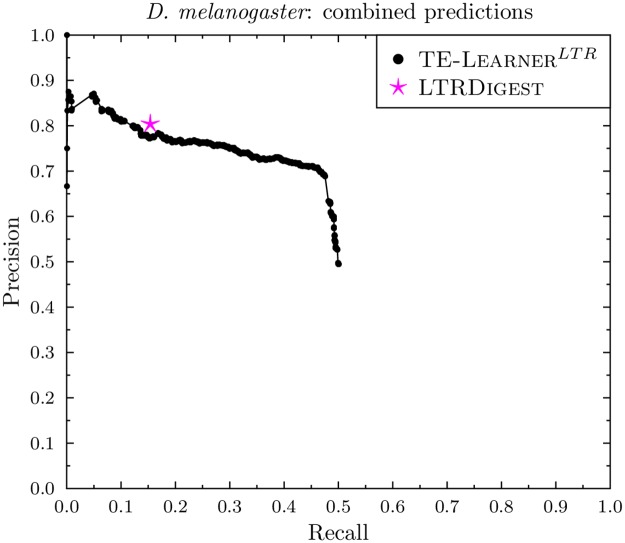
PR curve of the combined predictions in *D. melanogaster* for TE-Learner^*LTR*^ and LtrDigest.

**Fig 11:**
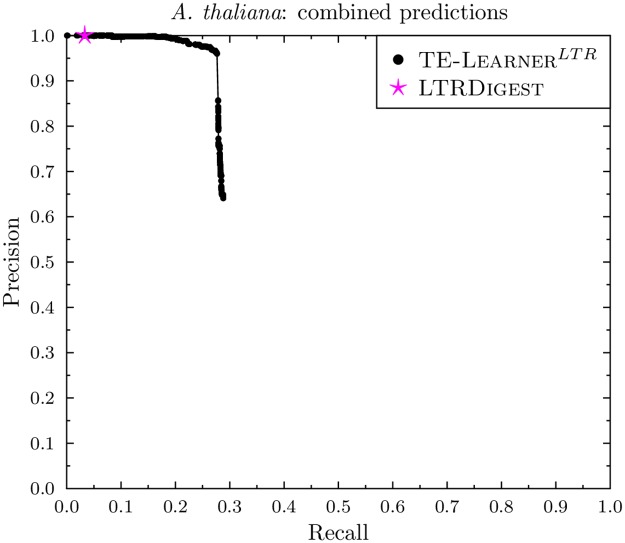
PR curve of the combined predictions in *A. thaliana* for TE-Learner^*LTR*^ and LtrDigest.

Figs 10 and 11 report the combined PR curves of TE-Learner^*LTR*^ and the point of LtrDigest. For *D. melanogaster*, the point of LtrDigest obtains a slightly higher precision (0.80) than TE-Learner^*LTR*^ (0.77) at a recall of 0.15. For *A. thaliana*, LtrDigest and TE-Learner^*LTR*^ both obtain a precision of 1, however, the latter obtains a higher recall. Moreover, our combined model has the advantage of allowing the user to choose an appropriate threshold.

## Discussion

To summarize the above analyses, first, TE-Learner^*LTR*^ outperformed LtrDigest in terms of recall, while it obtains a similar precision. Second, TE-Learner^*LTR*^ outperforms RepeatMasker and Censor in terms of precision, while the latter are able to obtain a higher recall. As we can see in Table 3, RepeatMasker and Censor make on average 3.7, resp. 14.2 times as many predictions as TE-Learner^*LTR*^ in order to obtain this recall, which comes at the cost of precision. Additionally, TE-Learner^*LTR*^’s lower recall can be explained by the fact that a considerable amount of transposable elements either do not have the protein domains considered in this study (see Table 1) or have domains that were not detected by RPS-Blast and are for these reasons not found by TE-Learner^*LTR*^. Third, the fact that TE-Learner^*LTR*^ and Censor output probabilities does give the advantage for a domain expert to choose a threshold based on the performance he or she prefers (either precision or recall).

In order to have an overview about how many TEs are found exclusively by each method, we created Table 4. It shows the number of annotations found by every method at the highest recall point, regardless of the precision. The table confirms that RepeatMasker and Censor find indeed more TEs than TE-Learner^*LTR*^, however, the latter is still able to find a significant number of TEs that the other methods were not able to find.

**Table 4:** Comparison of the number of annotations found by each method for each superfamily at the highest recall point. Column A indicates the number of annotations found exclusively by TE-Learner^*LTR*^, column I is the number of annotations that were predicted by both methods and column B indicates the number of annotations found exclusively by the compared method.

	*D. melanogaster*			*A. thaliana*		
Superfamily	A	I	B	A	I	B
TE-Learner^*LTR*^ vs. RepeatMasker						
Copia	6	165	11	49	596	351
Gypsy	82	1625	1072	114	1053	1404
Bel-Pao	5	344	183	–	–	–
TE-Learner^*LTR*^ vs. Censor						
Copia	2	169	11	50	595	366
Gypsy	81	1626	1065	104	1063	1399
Bel-Pao	11	338	172	–	–	–
TE-Learner^*LTR*^ vs. LtrDigest						
Copia	128	43	1	507	138	18
Gypsy	1293	414	46	1134	33	9
Bel-Pao	218	131	5	–	–	–

To conclude the above analysis, we computed the widely used F1 measure, which summarizes precision and recall in a single measurement. F1 corresponds to the harmonic mean of precision and recall, and measures the effectiveness of identifying TEs when giving equal importance to precision and recall. For the methods that output a PR *curve* (i.e., TE-Learner and Censor), we list the maximal F1 that can be obtained along the curve. The results, shown in Table 5, show that, on average, TE-Learner^*LTR*^ obtains the best F1-score. Only in 2 cases it was outperformed by RepeatMasker only; in all other cases it outperformed the other methods. Censor and LtrDigest are not competitive w.r.t. this evaluation measure. The F1 scores for the combined predictions confirm these results: on *D. melanogaster* we obtain 0.56 for TE-Learner^*LTR*^ versus 0.26 for LtrDigest, on *A. thaliana* the values are 0.43 and 0.06, respectively.

**Table 5:** F1-score of each method for each target organism and superfamily.

Target organism	*D. melanogaster*			*A. thaliana*		Avg.
Superfamily	Copia	Gypsy	Bel-Pao	Copia	Gypsy	
TE-Learner^*LTR*^	0.76	0.57	0.56	0.50	0.42	**0.56**
RepeatMasker	0.20	0.47	0.58	0.48	0.59	**0.46**
Censor	0.12	0.19	0.28	0.06	0.14	**0.16**
LtrDigest	0.34	0.24	0.36	0.16	0.02	**0.22**

On a MacBook Pro 2.8 GHz Intel Core i5, it took TE-Learner^*LTR*^ less than 10 minutes per superfamily to produce predictions from the *D. melanogaster* and *A. thaliana* genomes, including the annotation of the protein domains. LtrDigest runs as fast as TE-Learner^*LTR*^, but RepeatMasker and Censor have a much longer runtime: they are respectively 232 and 7 times slower than TE-Learner^*LTR*^ (averaged over the two target organisms and the three superfamilies).

Finally, since our method outputs probabilities and achieves high precision, it can be used to discover missing annotations: if a prediction receives a high score but is not in Flybase or Araport11, it may indicate a missing or incorrect annotation. To verify this, we have performed a BLAST search against the Nucleotide Collection (nt) of NCBI, for the top five false positive predictions for each superfamily and each target organism. Default search parameters were used. The results, shown in Table 6, confirm that for *D. melanogaster* all these predictions match with a high confidence to a retrotransposon hit, belonging to the respective superfamily. The only exception is the top two false positive predictions for Gypsy, which show a very low query coverage. Further inspection learned that all these annotations are indeed missing in Flybase, except the first false positive for Copia, which is in Flybase, but is annotated on the wrong strand. In general, we observed that 14 annotations for Copia in Flybase describe strands different of the strands retrieved by RPS-Blast for their conserved domains; for Gypsy and Bel-Pao, these numbers are 34 and 5, respectively. For *A. thaliana*, only three false positives lead to a TE hit, which belongs to the respective superfamily.

**Table 6:** BLAST analysis of the false positive (FP) predictions with the highest score. The first four columns show the location of the FP sequence, the last four columns show the details of the BLAST TE hit.

chrom.	start pos.	end pos.	strand	hit	FP cover	e-value	identity
*D. melanogaster*							
Copia							
3R	1354188	1356929	+	AF492763.1	65%	0.0	94%
3R	2252810	2259304	-	X02599.1	70%	0.0	99%
3R	1364299	1367899	-	X07656.1	100%	0.0	96%
2R	3956904	3960831	-	FJ238509.1	100%	0.0	99%
2L	23105894	23109919	-	X02599.1	100%	0.0	99%
Gypsy							
X	21872221	21878159	-	S68526.1	1%	3e-22	96%
2R	3790612	3798451	+	AY048125.1	0%	7e-10	89%
X	23476916	23480018	-	AF541949.1	100%	0.0	97%
X	23340454	23343541	-	X59545.1	100%	0.0	97%
3R	3675376	3680906	+	X14037.1	84%	0.0	99%
Bel-Pao							
3R	31890608	31894664	-	AY180917.1	100%	0.0	99%
3R	3035596	3042707	-	AY180917.1	68%	0.0	99%
3R	1657218	1661273	+	AY180917.1	100%	0.0	99%
3L	27623316	27627575	-	AJ487856.1	100%	0.0	99%
2R	4107747	4111802	+	AY180917.1	100%	0.0	99%
*A. thaliana*							
Copia							
5	19473171	19473572	-	NM_124179.2	100%	0.0	100%
3	15404097	15404507	-	XM_018601062.1	99%	4e-100	83%
Gypsy							
4	5080042	5082690	+	FJ197993.1	11%	5e-37	77%

### Conclusion

In this paper we have proposed a framework based on machine learning to identify and classify TEs in a genome. We evaluated our approach on three Class I TE superfamilies in *D. melanogaster*, and two Class I TE superfamilies in *A. thaliana*, using a relational random forest model. We found a better predictive performance (w.r.t. F1 measure) and runtime compared to three widely used methods for TE identification and classification. In terms of F1, the performance of RepeatMasker comes close to TE-Learner^*LTR*^, however, it obtains a higher recall, because it is able to recover TEs that have no conserved protein domains. The fact that we rely on these protein domains is a clear limitation of our method, yet, we are able to find TEs that other methods did not find. This suggests that TE-Learner^*LTR*^ presents a viable alternative to the state-of-the-art methods, in case one prefers predictions with a very high precision, or as a complement to the other methods when one is interested in finding more TEs. Furthermore, for our top predictions not confirmed by the official annotations, we validated their homology to known TEs of the corresponding superfamilies, showing that our method could be useful to detect missing annotations.

While our implementation has been focusing on LTR retrotransposons, it is possible to train it on other TE orders with superfamilies that have recognizable protein domains. Alternatively, one could change the implementation of any of the steps of the framework: the machine learning model, the features used, and the candidate generation procedure. For instance, to identify TEs from the TIR order (a Class II order with Terminal Inverted Repeats), the first step could use software tools to identify a candidate set of sequences surrounded by TIRs (such as [30, 31]).

A possible direction for further work is to explore hierarchical classification methods in the machine learning step of the framework. This would allow to exploit the underlying structure of the TE classification scheme. Additionally, one could try to still boost the performance of the different steps of the framework, e.g., by improving protein domain detection, or by including additional features (including features not related to protein domains) in the decision trees.
